# Small-Pox and Vaccination

**Published:** 1886-05

**Authors:** 


### Small-pox and Vaccination.

Perhaps nothing has occurred in recent times better calculated to impress minds of both the profession and the people with the inestimable value of vaccination as a reliable preventive of small-pox, than the unusual prevalence of the latter disease in the city of Montreal, having a considerable percentage of her population not only unvaccinated, but by a prejudice and want of knowledge stubbornly opposed to being vaccinated.—*Journal American Medical Association.*

A New Antiseptic.—Those of us who use iodoform in the consulting room must frequently have been seriously annoyed by its powerful and persistent smell. Drs. Silber and Ciammician, of Rome, have found an admirable substitute which has all the advantages of iodoform without its odor or, it is said, its poisonous properties. This substance is iodol, which occurs as a dark powder with a slight scent, reminding one of thymol. It is very slightly soluble, and is best used either in substance or suspended in glycerine, or made into an ointment with vaseline. A lotion can also be made by dissolving 1 grámme of iodol in 16 grammes of alcohol and adding 34 grammes of glycerine. Most brilliant results have been obtained by the use of the substance itself on chancres and syphilitic adenitis. In simple indolent ulcers, too, the use of the iodol lotion has been very beneficial. A spot of lupus on the leg was treated by injections of iodol solution into the surrounding subcutaneous tissue with the result of preventing the disease from spreading. Iodol has also proved useful in fungating joint diseases. Over 200 observations have been made, and neither erysipelas nor a diphtheritic condition of wounds has occurred.—*Wiener medicinisches Blatt.—Medical Times and Gazette*, December 5, 1885.

THE following case, in which nitrite of amyl was used as an antidote to opium, will doubtless be considered sufficiently important to lead others to try the same treatment in desperate cases of opium poisoning. The case recorded is of a person who took two ounces of laudanum, and showed every symptom of opium poisoning—coma, small pulse, feeble and infrequent respiration (six to the minute), coldness and cyanosis. Belladonna proved useless, while inhalation of nitrite of amyl immediately improved and ultimately restored the patient.

